# Immunotherapy for extensive-stage small-cell lung cancer: current landscape and future perspectives

**DOI:** 10.3389/fonc.2023.1142081

**Published:** 2023-04-28

**Authors:** Shuang Zhang, Ying Cheng

**Affiliations:** Department of Thoracic Oncology, Jilin Cancer Hospital, Changchun, China

**Keywords:** small cell lung cancer, immunotherapy, immune checkpoint inhibitors, biomarker, ES-SCLC

## Abstract

Small-cell lung cancer (SCLC) is a fatal subtype of lung cancer characterized by high aggressiveness, poor prognosis, and limited treatment options. For the first time in more than three decades, it has been demonstrated that the addition of immunotherapy to chemotherapy improved the survival of patients with extensive-stage SCLC, thereby immunotherapy plus chemotherapy established a new standard of first-line treatment. However, it is important to improve the curative effect of immunotherapy on SCLC and identify the patients who could benefit from such treatment. In this article, we review the current status of the first-line immunotherapy, the strategies to improve the efficacy of immunotherapy and the discovery of potential predictive biomarkers of immunotherapy for SCLC.

## Introduction

Small-cell lung cancer (SCLC) is highly malignant and aggressive subtype of lung cancer with dismal outcomes, which is closely related to tobacco exposure and accounts for approximately 15% of all lung cancers (1). SCLC is divided into a limited stage SCLC (LS-SCLC) and an extensive stage SCLC (ES-SCLC) according to the US Veterans Administration staging system (2). For decades, platinum-based chemotherapy has been the cornerstone of SCLC treatment. Despite dozens of randomized controlled trials, the efficacy of platinum plus etoposide has not been surpassed. Progression-free survival (PFS) is <6 months, and median overall survival (OS) is approximately 10 months (3, 4). As a “recalcitrant cancer,” SCLC urgently needs new treatment strategies.

The advent of immune checkpoint inhibitors has revolutionized the standard treatment options for many solid tumors including SCLC. The programmed death ligand-1 (PD-L1) inhibitor atezolizumab combined with chemotherapy improved survival with ES-SCLC in the Impower133 trial (5). The median OS of the atezolizumab group and placebo group was 12.3 and 10.3 months, respectively (hazard ratio [HR], 0.70; 95% confidence interval [CI], 0.54–0.91). Atezolizumab was also associated with a significant improvement in PFS (HR, 0.77; 95% CI, 0.62–0.9). The IMpower133 trial was the first to demonstrate a survival benefit of treatment with PD-L1 inhibitors in patients with ES-SCLC. The CASPIAN trial was a global, randomized, open-label phase III trial. Unlike the IMpower133 trial, the CASPIAN trial included three treatment groups (1:1:1 randomization): durvalumab plus chemotherapy group, durvalumab plus tremelimumab plus chemotherapy group, and chemotherapy group. A total of 805 patients were randomized in this trial, and OS was the primary endpoint. First-line treatment with durvalumab plus platinum–etoposide (EP) significantly improved the OS (HR, 0.73; 95% CI, 0.59–0.91) of patients with ES-SCLC. The median OS of patients in the durvalumab plus chemotherapy group and chemotherapy group was 13.0 and 10.3 months, respectively. However, there was no significant difference in PFS between these two groups (6). The trial met its primary endpoint. The results of the IMpower133 and CASPIAN studies confirmed that the PD-L1 inhibitor-chemotherapy strategy prolonged the OS of patients with ES-SCLC, while pembrolizumab, a programmed death-1 (PD-1) inhibitor targeting the same PD-1/PD-L1 signaling pathway, failed to improve OS of patients with ES-SCLC in the KEYNOTE-604 trial (7). KEYNOTE-604 was a randomized (1:1), double-blinded, phase III trial that investigated the efficacy and safety of pembrolizumab or placebo plus EP in patients with previously untreated ES-SCLC. This trial included 223 participants per group, and the primary endpoints were PFS and OS. A statistically significant improvement in PFS was reported (4.5 vs. 4.3 months, respectively; HR, 0.75 [95% CI, 0.61–0.91], *p*=0.0023). The median OS was 10.8 and 9.7 months, respectively (HR, 0.80; 95% CI, 0.64–0.98). However, the statistical threshold (*p*=0.0128) of significant prolongation of OS was not reached (*p*=0.0164). The KEYNOTE-604 study did not demonstrate significant improvement in survival following the addition of the PD-1 inhibitor pembrolizumab to standard chemotherapy. More research is needed to explore whether PD-1 inhibitors combination with chemotherapy can provide survival improved and to discover more effective treatment strategies for patients with ES-SCLC. It is not clear which patients will benefit from immunotherapy plus chemotherapy. In this review, we summarize completed and ongoing trials and discuss the current challenges and future research directions of immunotherapy for SCLC.

## New evidence of first-line immunotherapy for ES-SCLC

Recently, the results of two Phase III trials of first-line immunotherapy for ES-SCLC were published. One was the CAPSTONE-1 trial (8), which compared the efficacy and safety of the PD-L1 inhibitor adebrelimab (adebrelimab group) versus placebo in combination with chemotherapy (chemotherapy group) as first-line treatment in ES-SCLC. The primary endpoint was OS. Median OS of the adebrelimab group and the chemotherapy group were 15.3 and 12.8 months, respectively (HR, 0.72; 95% CI, 0.58–0.90; p = 0.0017). Adebrelimab plus chemotherapy significantly improved OS of patients with ES-SCLC. OS was numerically longer in both groups of the CAPSTONE-1 trial than that of the IMpower133 trial and the CASPIAN trial, which may be related to a higher proportion of patients receiving subsequent systemic treatments in the CAPSTONE-1 trial (59% and 70%) than other two trials (50.2% and 57.4% in the IMpower133 trial; 42% and 44% in the CASPIAN trial) (5, 6, 8). A lower proportion of patients with brain metastases included in the CAPSTONE-1 trial may also have contributed to this. However, the HR of OS in the CAPSTONE-1 trail was similar in the Impower133 trail (HR, 0.70) and the Caspian (HR, 0.73) trial. The CAPSTONE-1 trail further confirmed the results of the IMpower133 and CASPIAN trial, adding to the evidence for PD-L1 inhibitors plus chemotherapy. Adebrelimab plus chemoterapy is an alternate choice for first-line treatment of ES-SCLC.

The ASTRUM-005 trial (9) was another international, multicenter, phase III trial of the PD-1 inhibitor serplulimab or placebo plus chemotherapy for patients with ES-SCLC. The primary endpoint is also OS. Serplulimab plus chemotherapy prolonged OS of patients with ES-SCLC by 4.5 months and significantly reduced the risk of death (15.4 and 10.9 months, respectively; HR, 0.63; 95%CI, 0.49–0.82; p <0.001). The results still showed that serplulimab combination with chemotherapy favored PFS, objective response rate (ORR), and duration of response (DOR). This trial was the first to demonstrate that PD-1 inhibitor plus chemotherapy can also improve OS of ES-SCLC as first-line treatment, and the PD-1 inhibitor serplulimab in combination with chemotherapy is also an option for first-line treatment of ES-SCLC.

In addition, a 3-year survival rate was reported in the CASPIAN trial and KEYNOTE-604 trial. After a median follow-up of 39.4 months and 3.5 years, the 3-year survival rates of two trials were 17.6% and 15.5% in the immune-chemotherapy group, respectively, while the 3-year survival rates were 5.8% and 5.9% in the chemotherapy group of the two trials, respectively (10, 11), and the results were similar in the two trials. These results suggest that immunotherapy combination with chemotherapy can improve the long-term survival of patients with ES-SCLC. These results also support the addition of PD-L1 inhibitor to chemotherapy as the first-line treatment option for ES-SCLC.

Up to now, four drugs target on the PD-1/PD-L1 checkpoint in phase III trials have achieved positive results as the first-line immunotherapy of ES-SCLC. However, there is a lack of head-to-head comparison of efficacy and safety between different drugs. A randomized phase III trial to evaluate the activity and safety of serplulimab or atezolizumab plus chemotherapy as first-line treatment for patients with ES-SCLC is ongoing. Clinical Benefit Scale (CBS) is an objective and effective method for the evaluation of clinical benefit of drugs. It can be used as a reference for treatment selection in clinical practice by ranking the clinical benefit of multiple drugs in a certain type of treatment. Although direct comparisons between different trials need to be made with caution, when stratifying the expected benefits of the four trials about the first-line immunotherapy of ES-SCLC based on the European Society for Medical Oncology Magnitude of Clinical Benefit Scale (ESMO-MCBS) (12), the ASTRUM-005 trial was the only regimen with a score of 4. In addition, the short- and long-term efficacies of the ASTRUM-005 study were both improved, and the HRs of PFS and OS were the lowest among the four studies. The results were consistent with those of ESMO-MCBS. However, we also see that the median follow-up time of 12.3 months in the ASTRUM-005 trial is shorter than the median OS of 15.4 months in the serplulimab group (9), suggesting that the median OS, the HR for OS, and the 1- and 2-year OS rates may change as the follow-up time extension and the OS matures further. In terms of safety, durvalumab combination with chemotherapy had a lower incidence of grade ≥3 adverse events (AEs) and immunotherapy-related adverse events (irAEs), and serplulimab combination with chemotherapy had a lower incidence of treatment interruptions and treatment-related adverse events leading to death.

Although results from several phase III randomized controlled trials (RCTs) have established immunotherapy plus chemotherapy as a new standard of first-line treatment for ES-SCLC (**Table 1**). However, RCTs often exclude patients with active brain metastases, poor performance status, severe comorbidities, or autoimmune diseases. Whether immunotherapy plus chemotherapy is suitable for ES-SCLC in the real world also needs to be evaluated. A prospective real-world study of first-line immunotherapy for ES-SCLC from Spain included 155 patients (the Imfirst study) (13). There were 11.0% of patients with Eastern Cooperative Oncology Group performance status (ECOG) ≥2 and 17.4% of patients with brain metastases. The median PFS and OS of atezolizumab plus chemotherapy in the Imfirst study were 6.2 and 10.0 months, respectively. Of the patients, 27.8% and 4.5% of patients had the adverse events (AEs) and irAEs of grade ≥3, respectively, which were comparable to the Impower133 trial. A real-world retrospective study from China evaluated the efficacy and safety of immunotherapy plus chemotherapy versus chemotherapy alone (14). There were 14 (12.8%) patients and 11 (9.6%) patients with ECOG performance status≥2 in immunotherapy plus chemotherapy group and chemotherapy group. A total of 30 (27.5%) patients and 27 (23.5%) patients had brain metastases in two groups. A total of 27 (24.8%) patients and 33 (28.7%) patients had liver metastases, respectively. The median PFS was 8.5 and 5.0 months, and OS was 19.0 and 12.0 months, respectively. The proportion of patients with ≥ grade 3 AEs was similar between the two groups, and 32.1% of patients in the immunotherapy group had irAEs. This study suggests that immunotherapy plus chemotherapy can also prolong survival of patients with ES-SCLC in the real world, and it has a good safety profile. In contrast, another real-world prospective study of first-line chemoimmunotherapy for ES-SCLC investigated the efficacy and safety depending on whether the key eligible study criteria of previous trials are met (15). A total of 207 patients were enrolled in the study, of which 75 patients were ineligible for previous trials, including ECOG performance status 2–3, active brain metastases, uncontrolled pleural effusion, abnormal laboratory tests, immunosuppressive therapy, autoimmune diseases, and other malignant tumors within 5 years. The median PFS of eligible patients and ineligible patients were 5.1 and 4.7 months, respectively (HR, 0.72; 95% CI, 0.53–0.97, *p* =0.03). The median OS was 15.8 and 13.1 months, respectively (HR, 0.73; 95% CI, 0.51–1.07, *p* =0.10). Serious adverse events (SAEs) occurred in 27% and 39% of patients, respectively (*p* = 0.07). Although the OS of ineligible patients is similar to that of eligible patients, ineligible patients have a higher risk of SAEs. Therefore, it is still necessary to be cautious about the choice chemoimmunotherapy for some ineligible patients.

**Table 1:** Pivotal trials of the PD-L1/PD-1 inhibitor in combination with chemotherapy in ES-SCLC.

Trial Name	IMpower 133	CASPIAN		CAPSTONE-1	KEYNOTE-604	ASTRUM-005
**N**	201 vs. 202	268 vs. 269 vs. 269		230 vs. 232	228 vs. 225	389 vs. 196
**The primary outcome**	OS, PFS	OS		OS	PFS, OS	OS
**Brain metastases, N(%)**	17 (8.5%) vs. 18 (8.9%)	28 (10%) vs. 27 (10%) vs. 38 (14%)		5 (2%) vs. 5 (2%)	33 (14.5%) vs. 22 (9.8%)	50 (12.9%) vs. 28 (14.3)
**Liver metastases, N(%)**	77 (38.3%) vs. 72(35.6%)	108 (40%) vs. 104 (39%) vs. 117 (44%)		73 (32%) vs. 74 (32%)	95 (41.7%) vs. 92 (40.9%)	99 (25.4%) vs. 51 (26.0%)
**III stage, N (%)**	NA	28 (10%) vs. 24 (9%) vs. 18 (7%)		8 (3%) vs. 6 (3%)	0 vs. 0	NA
**ECOG 1, N(%)**	128 (63.7%) vs. 135(66.8%)	169 (63%) vs. 179 (67%) vs. 159 (59%)		197 (86%) vs. 202 (87%)	168 (73.7%) vs. 169 (75.1%)	318 (81.7%) vs. 164 (83.7%)
**Never smoking, N (%)**	9(4.5%) vs. 3(1.5%)	22 (8%) vs. 15 (6%) vs. 15 (6%)		50 (22%) vs. 53 (23%)	8 (3.5%) vs. 8 (3.6%)	81 (20.8%) vs. 35 (17.9%)
**Size of target lesions, median, mm in diameter**	113.0 vs. 105.5	NA		NA	134.8 vs. 126.6	117.7 vs. 120.5
**Treatment arms**	Atezolizumab + EC vs. Placebo + EC	Durvalumab+EC/EP vs. EC/EP	Durvalumab+ tremelimumab+ EC/EP vs. EC/EP	Adebrelimab+EC vs. placebo+EC	Pembrolizumab+EC/EP vs. Placebo+EC/EP	Serplulimab+EC vs. placebo+EC
**Median follow-up**	13.9m	14.2m	25.1m	13.5m	21.6m	12.3m
**OS (months; HR, 95% CI)**	12.3 vs. 10.3 0.70 (0.54–0.91)	13.0 vs. 10.3 0.73 (0.59–0.91)	10.4 vs. 10.5 0.82 (0.68–1.00)	15.3 vs. 12.8 0.72 (0.58–0.90)	10.8 vs. 9.7 0.80(0.64–0.98)	15.4 vs. 10.9 0.63(0.49–0.82)
**OS rate at 1 year**	51.7% vs. 38.2%	54% vs. 40%	43.8% vs. 39.3%	62.9% vs. 52.0%	45.1% vs. 39.6%	60.7% vs. 47.8%
**OS rate at 2 year**	NA	NA	23.4% vs. 14.4%	31.3% vs. 17.2%	22.5% vs. 11.2%	43.1% vs. 7.9%
**PFS, (months; HR, 95% CI)**	5.2 vs. 4.3 0.77 (0.62–0.96)	5.1 vs. 5.4 0.78 (0.65–0.94)	4.9 vs. 5.4 0.84 (0.70–1.01)	5.8 vs. 5.6 0.67 (0.54–0.83)	4.5 vs. 4.3 0.75 (0.61–0.91)	5.7 vs. 4.3 0.48(0.38–0.59)
**ORR**	60.2% vs. 64.4%	68% vs. 58%	58% vs. 58%	70.4% vs. 65.9%	70.6% vs. 61.8%	80.2% vs. 70.4%

## Gains and losses of PD-1 inhibitors as the first-line treatment in patients with ES-SCLC

At present, the results of several trials of PD-L1 inhibitors combination with chemotherapy are consistent, which can prolong the survival of patients with ES-SCLC. However, the effect on OS of the addition of PD-1 inhibitor to chemotherapy was different in two phase 3 trials of first-line treatment in ES-SCLC (**Table 1**). The inconsistent results may be related to the following factors. First, although both trials of untreated ES-SCLC, there were differences in the baseline characteristics of the patients enrolled. Liver metastases accounted for more than 40% in the KEYNOTE-604 trial (7) and 25% of patients in the ASTRUM-005 trial (9). In addition, in subgroup analyses of the KEY-NOTE-604 (7) and Impower133 trial (5), there was no trend toward a significant OS improvement for patients with liver metastases receiving immune combination chemotherapy compared with standard chemotherapy. The median sum of largest diameters of target lesions of immunotherapy group was 134.8 mm in the KEYNOTE-604 trial (7) vs. 117.7 mm in the ASTRUM-005 trial (9); patients in the immunotherapy group had a higher tumor burden in the KEYNOTE-604 trail (7). Furthermore, there was significant difference in age, sex, ECOG score, smoker history, and race between the two trials, which may have affected OS. Second, differences in the study design may also have contributed to the divergent results of OS in the two phase III trials. The KEYNOTE-604 trial had the dual primary end points for PFS and OS; two interim analyses and a final analysis were planned, and the two interim analyses were delayed, all of which led to the actual alpha consumption expected to be larger. In the end, the KEYNOTE-604 trial missed the positive result of OS with an extremely small difference. Third, subsequent cancer therapies were different between the two trials. In both trials, the continuation of the original immunotherapy regimen plus second-line standard chemotherapy after first-line treatment of progressive disease (PD) was allowed at the discretion of the investigator. In the ASTRUM-005 trial (9), 108 (27.8%) patients in the serplulimab group received checkpoint inhibitor rechallenge after PD, of whom 95 patients (24.4%) received serplulimab rechallenge. However, in the KEYNOTE604 trial (7), only nine patients (4%) in the pembrolizumab group received subsequent immunotherapy, of whom only one patient (0.4%) received pembrolizumab. In the OAK trial, the median post-PD OS was longer in patients who had PD who continued atezolizumab than in patients switching to non-protocol therapy (16). Recently, a retrospective study showed a significantly improved PFS of immunotherapy rechallenge in patients with ES-SCLC who progressed after first-line immunotherapy plus chemotherapy using inverse propensity weighting to balance baseline characteristics (4.8 vs. 2.4 m; HR, 0.40; 95%CI, 0.24–0.67; *p*=0.002) and had a trend toward prolonged OS (17.4 vs. 8.0; HR, 0.55; 95%CI, 0.29–1.04, *p*=0.098) (17). In the ASTRUM-005 trial, PFS2 was a secondary end point, and we look forward to subsequent reports of efficacy and safety results in patients who have been rechallenged with immunotherapy. Finally, although pembrolizumab and serplulimab are both PD-1 inhibitors, the epitopes selected by the two drugs are different, so they are two different macromolecules antibodies. There may be differences in efficacy between the two drugs, but clinical studies are needed to confirm.

## Strategies to improve the efficacy of first-line immunotherapy for ES-SCLC

Although immunotherapy combination with chemotherapy has brought survival improvement to patients with ES-SCLC, the improvement in OS was modest. Researchers are also exploring more effective treatment strategies. Immunochemotherapy plus “X” has become an important exploration direction. The efficacy and safety of immunochemotherapy plus CTLA-4 inhibitors for ES-SCLC was also explored in the CASPIAN trial (18). However, this treatment strategy did not increase efficacy, but rather increased toxicities. A phase II study is ongoing on the addition of QL1706 to first-line chemotherapy in ES-SCLC (NCT05309629) to determine whether dual antibodies targeting PD-1 and CLTA-4 can bring different results. The T-cell immunoreceptor with immunoglobulin and ITIM domain (TIGIT), an inhibitory receptor, is expressed by several immune cells including CD4+T cells, CD8+ T cells, and natural killer cells (19). Treatment with TIGIT inhibitors may enhance the antitumor effect of PD-1/PD-L1 inhibitors. The SKYSCRAPER-02 trial was a randomized, double-blinded, phase III trial, which compared tiragolumab (a TIGIT inhibitor) plus atezolizumab and EP with atezolizumab plus EP as first-line treatment for ES-SCLC (20). However, the addition of tiragolumab to chemo-immunotherapy did not confer additional survival benefit (OS, 13.6 vs. 13.6 months, respectively). This evidence suggests that chemo-immunotherapy remains the standard first-line treatment option for ES-SCLC. In addition, the LAG3 inhibitor IBI110 (NCT05026593) combined with sintilimab and chemotherapy is also being explored. However, it remains unclear for the immune escape mechanism that plays a dominant role in the immune microenvironment of SCLC. The expression of TIGIT and LAG3 in SCLC tumor tissues has not been fully studied. The current dual-immune combination strategy lacks reliable theoretical support. Marian L. Bur et al. found that most SCLCs have low expression of the major histocompatibility complex class I (MHC-I) antigen processing pathway-related genes (21). The low expression of MHC-I is also one of the reasons for poor response of PD-1/PD-L1 inhibitors in SCLC. Epigenetic silencing of MHC-I in SCLC may be mediated by lysine (K)-specific demethylase 1 A (KDM1A) who encoded lysine-specific demethylase 1(LSD1). LSD1 inhibitors can restore the expression of MHC-I (22) and potentiate responses to anti-PDL1 antibody for SCLC (23), which suggests that LSD1 inhibitors plus chemo-immunotherapy is a promising approach for ES-SCLC. Bispecific antibodies (BsAb) have attracted more and more attention because they can simultaneously build a bridge between target cells and functional molecules or cells and stimulate a directed immune response. DLL3 is highly expressed in most SCLC, which is a potential therapeutic target. Tarlatamab is a bispecific T-cell connector that targets CD3 and DLL3. Furthermore, tarlatamab is independent of MHC-I expression to induce immune responses. Tarlatamab showed good efficacy and safety with an ORR of 23% in a phase 1 study of relapsed SCLC (24). A phase 1b trial is evaluating the efficacy and safety of atezolizumab and chemotherapy plus tarlatamab in treatment-naive ES-SCLC.

Tumor angiogenesis is a functional component of the tumor microenvironment, interacting with the immune microenvironment to promote tumor growth. The addition of anti-angiogenesis agents to chemo-immunotherapy as a first-line treatment has improved survival in patients with non-small cell lung cancer (NSCLC) (25). SCLC is characterized by high vascularization (26, 27). High levels of vascular endothelial growth factor (VEGF) inhibit the maturation of dendritic cells (28) and promote the proliferation of regulatory T cells and myeloid-derived suppressor cells (29). Abnormal tumor vessels increase the interstitial fluid pressure inside the tumor to affect the infiltration of effector T cells. In addition, hypoxia in the tumor also promotes the differentiation of tumor-associated macrophages toward the M2-like phenotype, which is an immuno-suppressive phenotype (30). In a mouse model of SCLC, anti-PD-L1 therapy induced an PD-1/T-cell immunoglobulin and mucin domain 3 double-positive exhausted T-cell phenotype. This effect was diminished by the addition of anti-VEGF-targeted treatment. Compared with anti-PD-L1 or anti-VEGF monotherapy, treatment with anti-PD-L1 plus anti-VEGF inhibition significantly improved PFS and OS in a mouse model of SCLC (31). These results indicate that the dual inhibition of VEGF and PD-L1 may be an effective therapeutic strategy for SCLC. It is worth exploring whether the addition of an anti-angiogenesis agent to chemo-immunotherapy can further improve the efficacy of first-line treatment in ES-SCLC. Several phase II and III trials are evaluating the efficacy and safety of the addition of an anti-angiogenesis agent to chemo-immunotherapy for untreated ES-SCLC.

In the era of chemotherapy, the addition of thoracic radiotherapy (TRT) to ES-SCLC can improve the local control rate and 2-year survival rate (32). Several phase III trials of the first-line treatment of ES-SCLC with chemo-immunotherapy did not allow patients to receive TRT. In the era of immunotherapy, it is worth exploring the potential effects of the addition of TRT to chemo-immunotherapy on efficacy and safety. One strategy involves consolidation therapy with TRT plus immunotherapy after the completion of four to six cycles of chemotherapy (**Table 2**). The safety and preliminary efficacy of pembrolizumab plus TRT (45 Gy in 15 daily fractions) after induction chemotherapy for ES-SCLC or large-cell neuroendocrine cancer were assessed in a phase 1 trial (33). The results showed that dose-limiting toxicity did not occur during the 35-day period, while grade ≥3 AEs occurred in only 6% of patients. Concurrent treatment with pembrolizumab and TRT was well tolerated. The median PFS and OS were 6.1 and 8.4 months, respectively. These results are comparable to the historical data (PFS and OS were 4 and 8 months, respectively) obtained from a phase III study of consolidative TRT alone for ES-SCLC (32). Nevertheless, in another phase 1/2 study of consolidation therapy (i.e., ipilimumab and nivolumab with TRT) (33), the incidence rate of grade ≥3 TRAEs was 61.9%. The results of these two studies with small sample sizes are inconsistent. Therefore, further data from phase 2/3 studies on consolidation therapy combining TRT with immunotherapy are required. Another strategy is consolidation therapy combining TRT with immunotherapy after chemo-immunotherapy. A phase II, single-arm trial explored the efficacy and safety of adebrelimab plus chemotherapy and sequential TRT as first-line therapy for ES-SCLC (34). The study included 31 patients; of these patients, 10 received TRT and 24 received at least one dose of adebrelimab. The median PFS was 7.56 months, and ORR was 50%. The ORR of patients who received TRT was 80%. The incidence of grade ≥3 TRAEs was 51.6%. According to the preliminary results, the administration of chemo-immunotherapy followed by TRT warrants further study. In addition, there are also some studies exploring TRT and chemo-immunotherapy concurrent treatment (**Table 2**). Of course, it is important to explore the efficacy and safety of different modes of TRT (including conventional-dose division, low-dose TRT, SBRT, etc.) plus chemo-immunotherapy.

**Table 2:** Select ongoing trials with immunotherapy plus thoracic radiation therapy in ES-SCLC.

Trial	Phase	TRT Setting	Interventions	thoracic radiotherapy(TRT)	N	Primary end point
**NCT04402788** **(RAPTOR/NRG-LU007)**	Phase 2/3	Maintenance after atezolizumab + chemotherapy	atezolizumab vs. atezolizumab+ TRT	QD on days 1-5 during weeks 1-5 only	138	PFS and OS
**NCT04462276** **(TREASURE)**	Phase 2	Maintenance after atezolizumab + chemotherapy	atezolizumab vs. atezolizumab+ TRT	30 Gy in 10 fractions	104	OS
**NCT03923270**	Phase 1	Maintenance after Chemotherapy	TRT + Durvalumab vs. TRT+ Durvalumab + Tremelimumab vs. TRT+ Durvalumab +Olaparib	30 Gy in 10 fractions	25	Unacceptable SAEs; PFS(Phase IB)
**NCT04314297** **(AVATAR 2)**	Phase 2	Maintenance after Chemotherapy	Anlotinib+ Durvalumab at the end of TRT	NA	33	PFS
**NCT04472949** **(SAKK 15/19)**	Phase 2	Maintenance after EC+ Durvalumab	TRT + Durvalumab	39 Gy in 13 fractions	46	PFS
**NCT04728230** **(PRIO)**	Phase 1/2	Maintenance	Chemotherapy + Durvalumab+ Olaparib+ TRT	NA	63	AEs
**NCT04562337**	Phase 2	Maintenance	SHR1316+Chemotherapy →TRT+ SHR1316	NA	67	OS
**NCT05161533** **(CASPIAN-RT)**	Phase 2	Maintenance	EC/EP+ durvalumab × 4 cycles→durvalumab + hypofractionated TRT	Hypofractionated TRT beginning cycle 5 or 6 of durvalumab	50	PFS
**NCT05403723**	Phase 1	First-line induction therapy*	SBRT+ durvalumab+EC →durvalumab maintenance	30Gy in 5 fractions or 27 Gy in 3 fractions to the primary tumor site between cycle 2 and cycle 3	50	AEs
**NCT05092412**	Phase 2	First-line induction therapy	Low-dose TRT + durvalumab + EP/EC	15 Gy in 5fractions starting from Day 1 in the first cycle	30	PFS
**NCT05223647** **(TRIPLEX)**	Phase 3	First-line induction therap	Chemo-immunotherapy + TRT vs. Chemo-immunotherapy	30 Gy in10 fractions between 2nd and 3rd cycle	302	Change in 1-year OS
**NCT04951115**	Phase 2	First-line induction therap	SBRT+ Chemo-Immunotherapy	6 Gy of radiotherapy targeting multiple sites of intrathoracic disease on Days 1-5 of cycle 1	42	AEs and PFS
**NCT04622228** **(Match)**	Phase 2	First-line induction therap	LDRT+ EC/EP + atezolizumab	15 Gy in 5 fractions from Day 1- 5 in the first cycle	55	ORR

TRT, thoracic radiotherapy; LDRT, low-dose radiotherapy; SBRT, stereotactic body radiotherapy.

*Patients with SD or PD completed two cycles of durvalumab+EC.

Consolidation or maintenance therapy after four to six cycles of induction therapy have been explored to improve the efficacy of first-line treatment for ES-SCLC. The CheckMate-451 trial was a double-blinded phase III trial evaluating the efficacy and safety of nivolumab plus ipilimumab, nivolumab, or placebo as maintenance therapy following first-line chemotherapy for ES-SCLC (35). However, nivolumab monotherapy or nivolumab plus ipilimumab did not improve the OS of patients with ES-SCLC. A subgroup analysis showed that nivolumab monotherapy tended to improve OS in patients who initiated maintenance therapy within 5 weeks of the last dose of first-line chemotherapy. This result suggests that the timing and schedule of immunotherapy as maintenance therapy warrants further exploration. A phase II, single-arm study explored the efficacy and safety of nivolumab plus rucaparib—a poly (ADP-ribose) polymerase (PARP) inhibitor—as maintenance therapy in patients who response to first-line chemotherapy (36). In the interim analysis, the study included 20 patients with partial response to first-line chemotherapy. The PFS was 7.27 months from the initiation of induction therapy; the PFS of one patient was more than 23 months. The results suggest that nivolumab plus rucaparib as maintenance therapy may offer lasting benefits for some patients. These studies also will explore predictive biomarkers associated with response. Lurbinectedin is a novel chemotherapeutic agent with immunomodulatory properties and was conditionally approved by the FDA for second-line treatment of SCLC. A phase 3 trial is also comparing the efficacy and safety of atezolizumab versus maintenance atezolizumab plus Lurbinectedin as consolidation treatment in patients with ES-SCLC who did not have PD to induction therapy with atezolizumab plus chemotherapy.

## To explore patients who responded to immunotherapy

It is a key to achieve a breakthrough of immunotherapy for SCLC to guide therapy according to predictive biomarkers. Potential biomarkers associated with response to SCLC immunotherapy are also under investigation. The commonly used markers of PD-L1 expression and tumor mutation burden (TMB) did not significantly correlate with response to first-line immunotherapy for patients with ES-SCLC.

Recently, researchers have divided SCLC into four subtypes based on the expression of key transcription factors of ASCL1, NEUROD1, and POU2F3. Among them, SCLC-I subtype had higher expression of immune-related genes and more immune cell infiltration. In a retrospective analysis of the IMpower133 trial, patients with SCLC-I had a significant OS improvement with atezolizumab plus chemotherapy compared with other subtypes (HR, 0.566; 95% CI, 0.321–0.998) (37). Similarly, the retrospective analysis of the CASPIAN trial also obtained consistent results (38), which suggested that SCLC-I may be a predictive biomarker for immunotherapy of ES-SCLC; however, it needs to be confirmed by prospective studies.

MHC-I molecules are responsible for presenting antigenic peptides to CD8+T cells and play an important role in immune surveillance (39). In SCLC, using a multiplexed immunofluorescence assay, researchers found that patients with high expression of MHC-I had more infiltration of CD3+ T cells and CD45+/PD-L1+ immune cells in intratumoral regions than those with low or no expression of MHC-I (40). SCLC with high expression of MHC-I was characterized by low neuroendocrine differentiation and epithelial–mesenchymal transformation. This type represents a small proportion of patients with SCLC exhibiting long-term response to immunotherapy (40). According to the results, MHC-I expression may also be a promising predictive biomarker of immunotherapy in SCLC.

SCLC has neuroendocrine (NE) and non-neuroendocrine (non-NE) phenotypes. Non-NE SCLC highly expressed immune-related genes, which were related to immune response. In addition, NOTCH signaling is usually activated in non-NE SCLC, and notch-signaling-related genes can also predict the efficacy of immunotherapy for SCLC (41). One study found that RB1 plays an important role in the immune response, and tumors lacking RB1 showed reduced immune responses to various stimuli (42). Although RB1 is absent in the majority of SCLC, SCLC with non-NE phenotype and high YAP1 expression is often accompanied by RB1 protein expression (43). RB1 wild-type SCLC was also enriched in the SCLC-Y subtype (43). Recently, it has been found that patients with RB1 loss of function (LOF) score low or RB1 wild in transcriptional level benefit more from immunotherapy, while non-NE subtypes cannot predict the response to immunotherapy, suggesting that RB1 functional status may be a more relevant biomarker of immune response in SCLC (44).

At present, a subset of patients with SCLC benefits from immunotherapy from retrospective and small-sample exploration research; further exploration is required. These is a lack of reliable biomarkers for response to immunotherapy in SCLC. The lack of tissue samples limits our understanding of the tumor microenvironment of SCLC. This is one of the reasons responsible for the slow development of biomarkers of immunotherapy for SCLC. Analysis based on liquid samples may facilitate the study of biomarkers.

## Conclusions

Since the IMpower133 trial first demonstrated that immunotherapy combination with chemotherapy could improve the survival of patients with ES-SCLC, it has been further confirmed in subsequent several trials of phase 3 of PD-1/PD-L1 inhibitors plus chemotherapy in the first-treatment of ES-SCLC. Meta-analysis also supported that first-line immunotherapy could bring survival benefits to ES-SCLC. Immunotherapy combination with chemotherapy has replaced chemotherapy as the new standard care of first-line treatment for ES-SCLC. At present, the benefit of immunotherapy for ES-SCLC is still limited, especially as it is not cost effective. Exploring better therapeutic strategies and selecting patients who can benefit from immunotherapy are the main research directions of first-line immunotherapy for ES-SCLC. In addition, in trials of first-line immunotherapy for ES-SCLC, regardless of whether the median OS of immunotherapy is significantly better than that of chemotherapy, a higher proportion of patients in the immunotherapy group survived for more than 2 years. Immune checkpoint drugs have different response patterns and pharmacokinetic characteristics from cytotoxic drugs. Previous efficacy evaluation systems and criteria based on cytotoxic drugs are facing challenges. The restricted mean survival time (RMST) and other indicators in evaluating the efficacy of immunotherapy in ES-SCLC also need to be explored. Although RCTs and real-world studies have included some patients with brain metastases, due to the limited sample size, more studies are needed to guide clinical decision regarding the efficacy of immunotherapy in patients with brain metastases and the safety profile of brain radiotherapy during immunotherapy. In the current trials, ES-SCLC with liver metastasis does not benefit from immune plus chemotherapy, while chemo-immunotherapy plus anti-vascular therapy can improve survival for patients with liver metastasis in NSCLC. Whether patients with liver metastasis in SCLC also benefit from this treatment mode needs to be explored. An in-depth understanding of the characteristics of the immune microenvironment of SCLC is the key to achieve a leap forward for individualized immunotherapy strategy.

## Author contributions

Article concept: SZ and YC. Literature data collection: SZ. Writing—original draft: SZ and YC. Writing review and editing: SZ and YC. Supervision: YC. SZ and YC have read and agreed to the published version of the manuscript.
